# Friends and family matter Most: a trend analysis of increasing e-cigarette use among Irish teenagers and socio-demographic, personal, peer and familial associations

**DOI:** 10.1186/s12889-021-12113-9

**Published:** 2021-11-03

**Authors:** Joan Hanafin, Salome Sunday, Luke Clancy

**Affiliations:** TobaccoFree Research Institute Ireland (TFRI), FOCAS Institute, TU Dublin, Dublin, Ireland

**Keywords:** E-cigarettes, Teenagers, Gender, Peer smoking, Parental monitoring, ESPAD, Ireland

## Abstract

**Background:**

E-cigarette *ever-use* and *current-use* among teenagers has increased worldwide, including in Ireland.

**Methods:**

We use data from two Irish waves (2015, 2019) of the European School Survey Project on Alcohol and other Drugs (ESPAD) to investigate gender and teenage e-cigarette use (*n* = 3421 16-year-olds). Using chi-square analyses, we report changes in e-cigarette *ever-use, current-use*, and associated variables. Using multivariable logistic regression, we analyse the increase in e-cigarette use and socio-demographic, personal, peer and familial associations, focusing on gender differences.

**Results:**

E-cigarette *ever-use* increased from 23% in 2015 to 37% in 2019, and *current-use* from 10 to 18%. Compared with 2015, the odds in 2019, of becoming both an e-cigarette *ever-user* and *current-user*, were significantly higher for girls than boys (*ever-use*: AOR 2.67 vs 2.04; *current-use*: AOR 3.11 vs 1.96). Smoking and *e-cigarette use* are linked but never-smokers who try e-cigarettes rose significantly from 33 to 67% and those using e-cigarettes to quit smoking decreased significantly from 17 to 3%. Almost two-thirds of respondents (66%) in 2019 said that their reason for trying e-cigarettes was “out of curiosity”. Peer smoking is significantly associated with likelihood of e-cigarette *ever-use* (AOR 6.52) and *current-use* (AOR 5.45). If “Most/All friends smoke”, odds were significantly higher for boys than for girls (*ever-use* AOR 7.07 vs 6.23; *current-use* AOR 5.90 vs 5.31). Less parental monitoring is significantly associated with greater *e-cigarette ever-use* (AOR 3.96) and *current-use* (4.48), and having parents who usually don’t know where their child is on Saturday nights was also associated with significantly higher odds for boys than for girls (*ever-use* AOR 5.42 vs 3.33; *current-use* AOR 5.50 vs 3.50).

**Conclusion:**

Respondents had significantly higher odds of being e-cigarette ever- and current-users in 2019 compared with 2015. Use is higher among boys but girls are increasingly at risk. Two-thirds had never smoked cigarettes at first e-cigarette use; two-thirds used out of curiosity but few (3%) for smoking cessation. The most prominent risk factors for e-cigarette use were peer- and parent-related, especially so for boys. Interventions that take account of friend and family influences may provide mechanisms for preventing an increasing risk of nicotine addiction.

**Supplementary Information:**

The online version contains supplementary material available at 10.1186/s12889-021-12113-9.

## Background

E-cigarette use is increasing worldwide, and e-cigarettes are the most commonly used tobacco product among adolescents [1], including in Ireland [2]. A secondary analysis of five Irish health datasets (all stratified random samples in school-based settings) which included data on teenage tobacco use found that e-cigarette use has risen rapidly among adolescents in Ireland in recent years (from 23% *ever-use* in 2014 to 37% in 2019, and from 3% *current-use* in 2014 to 18% in 2019) [3].

Concerns about e-cigarettes at the level of public health and tobacco control have been aired for some time [4], including the effects of the mainstream tobacco industry’s entry into the e-cigarette marketplace. Smokefree legislation, at work and in recreational venues, has denormalised public cigarette smoking but the presence of e-cigarette aerosols in public view may tend to undo this situation [4–6].. Currently, concerns about e-cigarette harms include negative impact on cardiovascular health [7] and potential hazards such as obstructive lung disease from flavorants in e-cigarettes [8]. Passive exposure to second hand aerosol (SHA) from e-cigarettes is also a cause for concern, especially among those with respiratory diseases such as COPD [9, 10], and symptoms of sensory irritation, and general complaints have been reported by non-smokers [11].

Among adolescents, additional and specific potential negative consequences of e-cigarette use have been identified [12, 13]. There is “compelling” evidence that nicotine exposure during adolescence causes both long-term structural and functional changes in the brain with multiple adverse health consequences [14]. The series of risks identified from nicotine exposure include altered development of cerebral cortex and hippocampus in the developing adolescent brain [12]. The link between teenage e-cigarette use and increased smoking is widely accepted and supported by several reviews [15–17] but the possible mechanisms need to be explored further.

The upward trend in teenage *e-cigarette use* in Ireland has occurred against the historical backdrop of a two-decade downward trend in teenage cigarette smoking, down from 41% in 1995 to 14% in 2015 [18]. Between 2015 and 2019, at the same time that *e-cigarette use* was increasing rapidly, the downward trend in teenage current cigarette use came to a standstill, remaining at 14% overall in 2019 and, in fact, reversed for boys, among whom smoking prevalence rose significantly to 16% in 2019 [2]. The decline of *ever* cigarette smoking in Ireland seems to continue, albeit not at the level of statistical significance. Elsewhere, it has been suggested that e-cigarette users do not fit the traditional risk profile of cigarette smokers [19], pointing to a separate need to understand e-cigarette use.

Teenage e-cigarette users are more likely to be male gender, older, have higher amounts of pocket money, and report tobacco smoking-related characteristics (including regular and heavier smoking, and having peers who smoke) [20]. Gender differences in teenage smoking have been widely researched (e.g. [21]), but gender differences in teenage *e-cigarette use* are “relatively unknown” [1]. A recent review of literature on US adolescent e-cigarette use concluded that boys appear to have greater *e-cigarette use*, but “girls may be at increased risk if e-cigarettes are targeted to them, as it has been for cigarettes” [1]. The Irish data with its clear upward trend point to much higher levels of *e-cigarette use* among boys than among girls, but little is known about why this is so.

We examine socio-demographic, personal, peer and familial factors previously identified as being associated with e-cigarette use and having comparable data points in our two ESPAD waves. These factors include gender, cigarette smoking, truancy, peer smoking, household composition, parental monitoring, perceived family wealth, and maternal relationship., [2, 15–17, 22, 23].

## Methods

Data are drawn from two Irish waves (2015 and 2019) of the European School Survey Project on Alcohol and other Drugs (ESPAD) [2, 18], a cross-sectional survey carried out every 4 years that collects comparable data in about 35 European countries on substance use, including e-cigarette use.

All methods were carried out in accordance with relevant guidelines and regulations.

### Sample

In Ireland, in both the 2015 and 2019 waves of the ESPAD surveys, students were surveyed using a two-stage probability sampling technique: first at national level (a stratified random sample) and then at school level (systematic random sampling of classes). In the first stage at national level, a stratified random sampling of schools in Ireland was carried out. The schools were stratified according to geographic region and school type (secondary, vocational, community/comprehensive), religious affiliation (Roman Catholic, Church of Ireland, inter-denominational), gender (all-boys, all-girls, mixed), and school-level disadvantage status (Delivering Equality of Opportunity in Schools: DEIS vs. non-DEIS).

In the second phase, within schools in the sample, a systematic random sampling technique was used to identify the target sample of those born in 1999 (for the 2015 wave) and 2003 (for the 2019 wave), to produce a nationally representative sample of students who turned 16 years old during the year of data collection, as per ESPAD criteria. Only students present on the day of the survey were sampled, yielding a response rate of 86% for the 2015 sample and 80% for the 2019 sample. The total valid sample for this study was 3421 16-year-olds, comprising 1472 students (born in 1999) in the 2015 sample, and 1949 students (born in 2003) in the 2019 sample. Completed survey data were entered manually into SPSS v22 as they appeared in the survey. Full accounts of the sampling and data cleaning procedure have been reported elsewhere [2, 22]. All anonymised data are retained on a secure server and accessible through request to the corresponding author.

#### Variables

*The outcome variables were ever-use and current-use of e-cigarettes.*

*The independent/exposure variables were socio-demographic, personal, peer, and familial characteristics, measured as described below.*

### Measurement

A full list of the questions, and answer categories, from which variables were drawn is in Additional files 1 & 2 (ESPAD questionnaires 2015 and 2019).

#### Ever-use and current-use of e-cigarettes

Two variables measured prevalence, *e-cigarette ever-use* and *e-cigarette current-*use.

*E-cigarette ever-use* was assessed by the question ‘Have you ever used e-cigarettes?’: Response options were: ‘Yes, more than 12 months ago; Yes, in the last 12 months; Yes, in the last 30 days; Never’, recoded ‘*ever-use*’ -no vs yes).

*E-cigarette current-use* was assessed by the question ‘How often have you smoked e-cigarettes during the last 30 days?’: ‘Not at all; Less than once per week; At least once a week; Almost every day’; recoded ‘*current-use*’ -no vs yes.

*Socio-demographic variables*includedgender (male, female), perceived wealth (recoded much better off, better off, about the same, less well off) and household composition (one-parent, two-parent, blended).

#### Personal, peer, and familial variables

Students were asked about:

*Their cigarette use* (2 measures: ever-smoked and current smoking).

Ever-smoked: ‘On how many occasions in your lifetime have you smoked cigarettes (excluding e-cigarettes)?’; Response options were: ‘Number of occasions: 1–2, 3–5, 6–9, 10–19, 20–39, 40 or more’, recoded ‘ever-smoked’ -no vs yes.

Current smoking: ‘How often have you smoked cigarettes (excluding e-cigarettes) during the last 30 days?’; ‘Not at all; less than 1 cigarette per week; less than 1 cigarette per day; 1–5 cigarettes per day; 6–10 cigarettes per day; 11–20 cigarettes per day; more than 20 cigarettes per day’, recoded ‘current smoking’ -no vs yes.

#### Truancy

Respondents reported the number of days they had skipped or “cut” school in the previous 30 days (recoded none, 1–4 days, 5+ days).

#### Peer smoking

Respondents reported how many of their friends smoke cigarettes, (‘none’, ‘a few’, ‘some’, ‘most’ and ‘all’; recoded none, a few/some, most/all).

#### Parental monitoring

Respondents reported whether their parents (mother or father) know where they spend their Saturday nights (‘know always’, ‘know quite often’, ‘know sometimes’, ‘usually don’t know’).

#### Relationship with mother

Respondents reported their satisfaction with their relationship with their mother (recoded as satisfied, neither nor, not satisfied).

We also show sample characteristics for two additional variables (*Reasons for trying e-cigarettes*: To quit smoking; because friends were using it; out of curiosity) and *Relationship with tobacco when first tried an e-cigarette*: Never smoked tobacco; smoked occasionally; smoked regularly) but these were not included in the multiple regression analysis because the questions’ answer structure was not comparable across both waves.

### Statistical analysis

First, Pearson’s chi-square test was used to compare the demographic, personal, peer and familial variables between 2015 and 2019 and to determine whether differences in the variables between the two waves were statistically significant (Table 1). Then, e-cigarette *ever-use* and *current-use* were examined using a multivariable logistic regression model with *e-cigarette ever-use* (Table 2) and *current-use* (Table 3) as the dependent variable, analysed for all and by gender. To assess potential multicollinearity, all variables in the study were adjusted by the dependent variable (e-cigarette ever- and current-use) using Spearman’s correlation coefficient and variance inflation factor (VIF) as appropriate between variables, and a VIF < 5 was used to detect multicollinearity. All analyses and results are presented as adjusted odds ratios (AOR) and 95% confidence intervals. A *p*-value of .05 was used as a cut-off for significance. All statistical analyses were conducted using STATA version 16.

**Table 1:** Sample characteristics – crosstabulations of socio-demographic, personal, familial, and peer variables in 2015 and 2019 Irish ESPAD Survey Data

	ESPAD Year (2015 and 2019) and significance level^a^		
	2015 (*n* = 1472) n (%)	2019 (*n* = 1949) n (%)	*p-*value
*Gender*			
Male	752 (51.1)	946 (48.5)	
Female	720 (48.9)	1003 (51.5)	.140
*E-cigarette ever-use*			
Yes	**325 (23.0)**	**754 (39.0)**	
No	**1088 (77.0)**	**1219 (62.7)**	<.001
*E-cigarette current-use*			
Yes	**143 (10.1)**	**351 (18.1)**	
No	**1270 (89.9)**	**1592 (81.9)**	<.001
*Ever-smoked*			
Yes	473 (32.3)	614 (31.6)	
No	992 (67.7)	1328 (68.4)	.678
*Current smoking*			
Yes	191 (13.0)	281 (14.4)	
No	1275 (87.0)	1664 (85.6)	.235
*Household composition*			
Single parent	**262 (17.8)**	**371 (19.0)**	
Two parents	**1109 (75.3)**	**1490 (76.4)**	
Blended families	**101 (6.9)**	**88 (4.5)**	.010
*Parental Monitoring*^b^			
Know always	906 (62.7)	1194 (63.2)	
Know quite often	337 (23.3)	455 (24.1)	
Know sometimes	128 (8.9)	166 (8.8)	
Usually don’t know	73 (5.1)	74 (3.9)	.452
*Skipping School*			
None	984 (80.1)	1309 (79.6)	
1–4 days	198 (16.1)	286 (17.4)	
5 days+	47 (3.8)	50 (3.0)	.371
*Perceived family wealth*			
About the same	**696 (48.7)**	**815 (43.3)**	
Much better off	**223 (15.6)**	**308 (16.4)**	
Better off	**370 (25.9)**	**580 (30.8)**	
Less well off	**141 (9.9)**	**179 (9.5)**	.006
*Peers who smoke*			
None	478 (33.4)	558 (29.8)	
A few/some	802 (56.1)	1125 (60.1)	
Most/all	150 (10.5)	188 (10.1)	.056
*Relationship with mother*			
Satisfied	1251 (87.5)	1621 (86.4)	
Neither nor	74 (5.2)	106 (5.6)	
Not satisfied	105 (7.3)	150 (8.0)	.640
*Reasons for trying e-cigarettes*			
To quit smoking	**51 (17.3)**	**16 (3.4)**	<.001
Because friends were using it	**63 (21.4)**	**137 (28.8)**	<.001
Out of curiosity	**186 (63.1)**	**315 (66.3)**	<.001
*Relationship with tobacco when first tried e-cigarette*			
Never smoked tobacco	**101 (33.3)**	**461 (66.7)**	
Smoked tobacco occasionally	**155 (51.8)**	**168 (24.3)**	
Smoked tobacco regularly	**45 (14.9)**	**62 (9.0)**	<.001

^a^***Bold numbers indicate statistical significance at <0 .05***

^b^*Parents know where child is on Saturday nights*

**Table 2:** Socio-demographic, personal, peer and familial variables associated with *e-cigarette ever-use* in the 2015 & 2019 ESPAD Surveys: multivariable logistic regression

	E-cigarette ever-use (Adjusted Odds Ratio (AOR), total sample and by gender)		
	Total (AOR, 95% CI)	Boys (AOR, 95% CI)	Girls (AOR, 95% CI)
*Gender*			
Male	1		
Female	0.99 (0.83, 1.20)	N/A	N/A
*ESPAD Year*			
2015	1	1	1
2019	**2.29 (1.89, 2.78)**	**2.04 (1.55, 2.68)**	**2.67 (2.02, 3.54)**
*Ever smoked*			
No	**1**	1	1
Yes	**1.39 (1.10, 1.75)**	1.23 (0.89, 1.71)	**1.56 (1.12, 2.18)**
*Current smoking*			
No	**1**	1	1
Yes	**1.76 (1.31, 2.38)**	**2.60 (1.71, 3.93)**	1.14 (0.73, 1.79)
*Household composition*			
Single parent	1	1	1
Two parents	0.84 (0.66, 1.08)	0.91 (0.63, 1.30)	0.80 (0.55, 1.15)
Blended families	1.43 (0.93, 2.16)	**1.85 (1.02, 3.35)**	1.14 (0.61, 2.13)
*Parental Monitoring*			
Know always	1	1	1
Know quite often	**1.99 (1.61, 2.46)**	**1.94 (1.43, 2.62)**	**2.04 (1.51, 2.75)**
Know sometimes	**3.12 (2.52, 4.63)**	**3.15 (2.06, 4.83)**	**3.68 (2.35, 5.75)**
Usually don’t know	**3.96 (2.54, 6.18)**	**5.42 (2.72, 10.79)**	**3.33 (1.84, 6.03)**
*Skipping School*			
None	1	1	1
1–4 days	0.86 (0.67, 1.10)	0.83 (0.58, 1.18)	0.90 (0.64, 1.27)
5 days+	1.56 (0.95, 2.56)	**2.17 (1.08, 4.36)**	1.07 (0.50, 2.30)
*Perceived wealth*			
About the same	1	1	1
Much better off	1.11 (0.85, 1.45)	1.30 (0.88, 1.91)	0.96 (0.66, 1.40)
Better off	1.01 (0.81, 1.26)	1.31 (0.96, 1.79)	0.80 (0.59, 1.09)
Less well off	1.29 (0.93, 1.78)	0.94 (0.59, 1.51)	**1.76 (1.11, 2.78)**
*Peers who smoke*			
None	1	1	1
A few/some	**2.74 (2.17, 3.45)**	**3.12 (2.22, 4.38)**	**2.47 (1.79, 3.41)**
Most/all	**6.52 (4.66, 9.15)**	**7.07 (4.33, 11.55)**	**6.23 (3.87, 10.02)**
*Maternal relationship*			
Satisfied	1	1	1
Neither nor	1.65 (1.13, 2.40)	2.22 (1.33, 3.72)	1.55 (0.65, 2.04)
Not satisfied	1.36 (0.98, 1.89)	1.36 (0.85, 2.18)	1.32 (0.83, 2.10)

***Bold numbers indicate statistical significance at < .05***

**Table 3:** Socio-demographic, personal, peer and familial variables associated with *e-cigarette current-use* in the 2015 & 2019 ESPAD Surveys: multivariable logistic regression

	E-cigarette *current-use* Adjusted Odds Ratio (AOR) (total sample and by gender)		
	Total (AOR, 95% CI)	Boys (AOR, 95% CI)	Girls (AOR, 95% CI)
*Gender*			
Male	1		
Female	1.03 (0.81,1.30)	N/A	N/A
*ESPAD Year*			
2015	1	1	1
2019	**2.41 (1.85, 3.12)**	**1.96 (1.37, 2.82)**	**3.11 (2.10, 4.61)**
*Ever smoked*			
No	1	1	1
Yes	1.14 (0.84, 1.55)	1.19 (0.77, 1.82)	1.13 (0.72, 1.76)
*Current smoking*			
No	1	1	1
Yes	**1.78 (1.23, 2.55)**	**2.13 (1.30, 3.51)**	1.50 (0.87, 2.59)
*Household composition*			
Single parent	1	1	1
Two parents	0.94 (0.68, 1.29)	1.16 (0.72, 1.84)	0.75 (0.48, 1.62)
Blended families	1.46 (0.89, 2.44)	1.53 (0.72, 3.24)	1.37 (0.68, 2.76)
*Parental Monitoring*			
Know always	1	1	1
Know quite often	**2.22 (1.69, 2.92)**	**2.62 (1.76, 3.90)**	**1.90 (1.29, 2.81)**
Know sometimes	**3.53 (2.49, 5.01)**	**4.06 (2.49, 6.63)**	**3.09 (1.85, 5.15)**
Usually don’t know	**4.48 (2.83, 7.11)**	**5.50 (2.85, 10.61)**	**3.50 (1.79, 6.84)**
*Skipping School*			
None	1	1	1
1–4 days	0.86 (0.63, 1.18)	0.68 (0.42, 1.10)	1.03 (0.68, 1.58)
5 days+	1.42 (0.81, 2.51)	1.08 (0.49, 2.37)	1.70 (0.74, 3.90)
*Perceived wealth*			
About the same	1	1	1
Much better off	1.14 (0.82, 1.59)	1.04 (0.65, 1.67)	1.20 (0.75, 1.93)
Better off	0.77 (0.57, 1.03)	0.81 (0.53, 1.22)	0.73 (0.48, 1.11)
Less well off	1.28 (0.87, 1.88)	1.26 (0.72, 2.20)	1.35 (0.79, 2.33)
*Peers who smoke*			
None	1	1	1
A few/some	**2.13 (1.54, 2.96)**	**2.23 (1.39, 3.59)**	**2.11 (1.34, 3.33)**
Most/all	**5.45 (3.65, 8.14)**	**5.90 (3.31, 10.52)**	**5.31 (3.01, 9.37)**
*Relationship with mother*			
Satisfied	1	1	1
Neither nor	1.23 (0.77, 1.97)	1.37 (0.68, 2.79)	1.25 (0.66, 2.37)
Not satisfied	**1.55 (1.06, 2.26)**	1.40 (0.79, 2.49)	1.64 (0.98, 2.74)

***Bold numbers indicate statistical significance at < .05***

## Results

### Changes in prevalence of e-cigarette use between 2015 and 2019

**Fig. 1:**
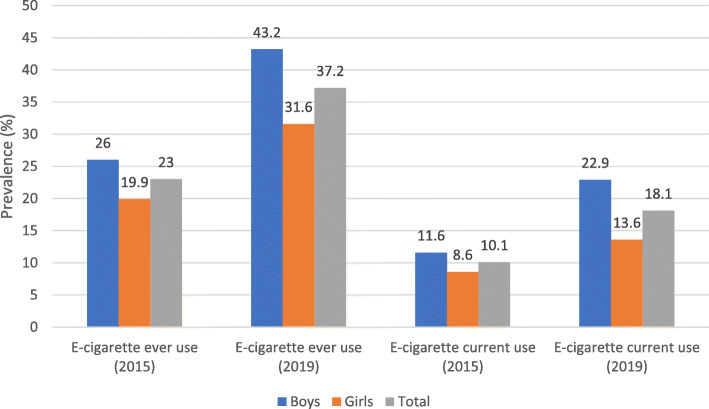
Changes in % prevalence of e-cigarette ever-use and current-use between 2015 and 2019 (Boys, Girls, Total). Source: ESPAD Ireland Survey Data 2015 and 2019

Prevalence of e-cigarette *ever-use* and *current-use* among Irish 16-year-olds rose significantly between 2015 and 2019. Prevalence of both is significantly higher for boys than for girls in both waves (Fig. 1). The overall prevalence of *ever-use* of e-cigarettes increased from 23 to 37%, with boys increasing from 26 to 43.2% and girls from 19.9 to 31.6%. *Current-use* increased overall from 10.1 to 18.1%, from 11.6 to 22.9% in boys and from 8.6% to 13.6 in girls. Most of the other factors included in our model are statistically unchanged between 2015 and 2019 (Table 1).

### Sample differences between 2015 and 2019

Pearson’s chi-square analyses (Table 1) of risk factors associated with e-cigarette use (socio-demographic, personal, peer and familial characteristics) show very few statistically significant differences (*p* < .05) between the 2015 and 2019 ESPAD waves.

Notable findings concern the relationship with tobacco use when first using e-cigarettes and reasons reported for trying e-cigarettes. In 2015, 33.3% had never used tobacco when they first used e-cigarettes and this rose to 66.7% in 2019. Also, the number who said that they were regular tobacco users when they first used an e-cigarette decreased from 14.9 to 9% between waves.

Reasons for trying e-cigarettes also changed, with significantly fewer reporting trying e-cigarettes in order *to quit smoking* in 2019 (3.4%) than in 2015 (17.3%). Trying e-cigarettes *out of curiosity* increased from 63.1% in 2015 to 66.3% in 2019, and those who used it *because of friend*s increased from 21.4% in 2015 to 28.8% in 2019.

### Trends in e-cigarette ever-use

Table 2 shows the results of the multivariable logistic regression analysis of e-cigarette ever-use in the 2015 and 2019 ESPAD Ireland surveys, reported as adjusted odds ratios (AOR, 95% CI) for the total sample, and separately for boys and girls.

The odds of *ever-using e-cigarettes* increased significantly for the whole sample between 2015 and 2019 (AOR 2.29, 95% CI:1.89–2.78), and the increase was more pronounced for girls (AOR 2.67, 95% CI:2.02–3.54) compared with boys (AOR 2.04, 95% CI:1.55–2.68). Use of combustible tobacco (both ever and current smoking) was associated with increased odds of *ever-using e-cigarettes,* with ever-smoking having higher odds for girls (AOR 1.56, 95% CI:1.12–2.18) and current smoking higher odds for boys (AOR 2.60, 95% CI:1.71–3.93).

Girls who reported that their families were less well-off than other families had increased odds (AOR 1.76, 95% CI:1.11–2.78) of being *ever-users* of e-cigarettes. Boys living in blended families also had increased odds (AOR 1.85, 95% CI:1.02–3.35).

Peer smoking was strongly associated with *e-cigarette ever-use*. Those who reported that “most or all” of their friends smoked had increased odds of ever-using e-cigarettes (AOR 6.52, 95% CI:4.66–9.15) and this was more pronounced for boys (AOR 7.07, 95% CI:4.33–11.55) than for girls (AOR 6.23, 95% CI:3.87–10.02).

Parental monitoring was significantly associated with ever-use for all (AOR 3.96, 95% CI:2.54–6.18), and was more important for boys (AOR 5.42, 95% CI:2.72–10.79) compared with girls (AOR 3.33, 95% CI:1.84–6.03). Truancy and being in a blended family were associated with increased odds for boys but not for girls.

### Trend in e-cigarette current-use

Table 3 shows the results of the multivariable logistic regression analysis of e-cigarette current-use in the 2015 and 2019 ESPAD Ireland surveys, reported as adjusted odds ratios (AOR, 95% CI) for the total sample, and separately for boys and girls.

The odds of teenagers being *e-cigarette current-users* increased significantly between 2015 and 2019 (AOR 2.41, 95% CI:1.85–3.12); the increase was greater for girls (AOR 3.11, 95% CI:2.10–4.61) compared with boys (AOR 1.96, 95% CI: 1.37–2.82). Being a current smoker increased the odds of *current-use e-cigarette* (AOR 1.78, 95% CI:1.23–2.55), significantly so for boys (AOR 2.13, 95% CI:1.30–3.51). The more friends young people have who smoke, the greater their odds of *e-cigarette current-use.* Those who reported that “most or all” of their friends smoked had greater odds of *e-cigarette current*-*use* (AOR 5.45, 95% CI:3.65–8.14), and this was more pronounced for boys (AOR 5.90, 95% CI:3.31–10.52) than for girls (AOR 5.31, 95% CI:3.01–9.37).

As with *ever-use* of e-cigarettes, decreased parental monitoring was also associated with increased odds for teenagers’ *current-use* of e-cigarettes. The odds were significantly increased (AOR 4.48, 95% CI:2.83–7.11) in young people who say that their parents “usually don’t know” where they are on a Saturday night (reference group “parents always know”). Odds were higher for boys (AOR 5.50, 95% CI:2.85–10.61) than for girls (3.50, 95% CI:1.79–6.84).

## Discussion

Results from our two ESPAD waves comprising 3421 16-year-olds show that *e-cigarette ever-use and current-use* increased significantly between 2015 and 2019 in Ireland. There was a significant rise in never-smokers trying e-cigarettes, with an increase from one-third (33%) to two-thirds (67%) of the sample who had never used tobacco when they first tried an e-cigarette. The link between cigarette and e-cigarette use in teenagers is clear but the mechanisms are uncertain. The longstanding Gateway Theory [24] of the centrality of nicotine addiction in the progression to other drugs is insufficient to explain fully the progression to cigarettes from e-cigarettes, especially as cigarette use often precedes e-cigarettes. The Common Liability Theory [25] allows for wider inputs from environmental and genetic influences, while the Catalyst Model [26] helps consider the factors influencing initiation and progression, which could possibly extend to a diversion model preventing progression to smoking [27]. Our finding of a marked increase in e-cigarette use in association with peer cigarette smoking allows for the possibility that a catalyst effect occurred but does not exclude the possibility of some “diversion” occurring, perhaps resulting in less progression to smoking in girls [28].

### Gender differences in e-cigarette use

From the outset, boys in our trend analyses were more likely to be both *ever-* and *current- users* of e-cigarettes. This is in line with many other studies [1, 20]. Various theories have been offered to explain gender and substance use including tobacco and e-cigarettes, such as Connell’s (2005) influential construct of hegemonic masculinity and how it puts men at risk of harmful health behaviours and consequences that can be destructive for them [29], including for teenage boys [30, 31], and Butler’s [32] consequential theory of gender performativity - that gender is not an essential, biologically determined quality or an inherent identity, but is repeatedly performed, based on, and reinforced by, societal norms, this repeated performance of gender being also performative - applied to smoking by women in Australia by Gilbert and colleagues [33]. They argued that smoking is “a gender act that can be internalised and which, when repeatedly performed by women in gender-appropriate ways, constructs a ‘feminine’ gender identity” [32, 33]. Such theories and how they relate to our findings on gendered e-cigarette use are outside the scope of our data. We raise them here to acknowledge that our findings have a broader and deeper context within discourses on gender and substance use [31].

Boys have higher prevalence of *e-cigarette use* but the rate of increase in this study is significantly greater for girls, and this was particularly pronounced for *current-use*, with the trend analysis showing girls having more than 50% higher odds (AOR 3.11, 95% CI 2.10–4.61) than boys (AOR 1.96, 95% CI 1.37–2.82) of being *e-cigarette current-users* in 2019 compared with 2015. This gendered pattern of substance use showing initial high male use, with female use subsequently over-taking that of males reflects historical patterns of women’s and men’s tobacco use, driven, in part at least, by the tobacco industry’s gendered marketing, and exploitation of social change and social disruption [34–36], such as the post-war targeting of women by the tobacco industry “as an equality and freedom issue” [36]. The latter comprised advertising and marketing by the industry, specifically and successfully targeted to women and girls, a market identified as a large untapped lucrative reservoir [34–36]. E-cigarette advertising and direct and covert marketing uses strikingly similar techniques to those used previously by the cigarette industry [37] - featuring young, attractive models, sponsorship of sports events and parties, product placement, and direct payments to social media influencers [37]. We add support to Kong et al. (2017) who observed that, while boys in the U.S. appear to have greater use of e-cigarettes, girls may be at increased risk if e-cigarettes are targeted to them “as it has been for cigarettes” and we join in calling for further research on gender differences in e-cigarette use, particularly in gendered rates of increase, and on the role of industry advertising and marketing, including the gendered nature of such activities on the internet [1]. We recommend that insights about gender, from emerging theories and historical developments such as those mentioned above, be incorporated into both policy-making and health education programmes that are intended to reduce children’s e-cigarette use.

We agree with O’Leary et al. (2019) that, while the state and use of social media are ever changing, the potential to use social media as a form of promotion for healthy behaviours, especially among adolescents, will continue to offer promise [38]. Thus, we extend to the domain of tobacco and e-cigarette use their call for education interventions for teenagers [38].

Also, we draw attention to findings from ourselves and others regarding the potentially different online worlds inhabited by teenage girls (social media platforms) and teenage boys (gaming platforms) that have been identified [2, 39]. This leads us to speculate, for example, that boys may be targeted through gaming platforms and that girls’ rapidly increasing *e-cigarette use* may be related to their greater social media use. The scope within these parallel gendered domains for targeted marketing of e-cigarettes by industry merits further research and we also support calls for regulatory action to prohibit sponsored e-cigarette content on social media platforms used by youth [40, 41].

### E-cigarette use and smoking

The link between cigarette smoking and *e-cigarette use* has been well-established [15–17] and our findings support this, but with gender differences. Girls who had ever-smoked had higher odds (AOR 1.56, 95% CI: 1.12–2.18) of *ever-using e-cigarettes*. Boys who were current smokers had more than twice the odds of being *e-cigarette ever-users* (AOR 2.60, 95% CI: 1.71–3.93). Thus, differences in experimentation and continuation of both smoking and e-cigarette use appear to be gendered, pointing to different characteristics between the cigarette-smoking and *e-cigarette using* populations or to gender differences that require further exploration. We lend some support to the findings of Creamer et al. (2021) that, regarding psychosocial risk factors for cigarette smoking, *e-cigarette users* do not fit the traditional risk profile of cigarette smokers, and also require further research [19].

### Peer influence

Adolescent peer social networks have been found to be important for health behaviour choices, with health behaviour similarity found to be driven by homophilic social selection and/or social influence [42]. Studies of adolescent social networks, including online networks, suggest that friends’ online behaviours are a viable source of peer influence [23]. Those with more peers who smoke have much higher odds of being *ever-users* of e-cigarettes, and this pattern was particularly strong for boys. Peer smoking was similarly implicated in *e-cigarette current-use* and, again, gender differences showed a somewhat stronger influence of peers on boys than on girls in relation to *current-use of e-cigarettes*. A review of 26 studies examining adolescents’ susceptibility to peer pressure to engage in risky behaviours identified two primary trends: one, that adolescent males appear to be more susceptible to peer influences that encourage risk-taking behaviours; and the other, that there are no consistent gender differences [43]. McCoy and colleagues conjecture [43] that, as attitudes about gender-appropriate behaviour shift across historical time, it may be that male and female teenage experiences are becoming increasingly similar, for example in experiencing comparable levels of deviant peer pressure around substance use in particular and also that differences across types of risky behaviours may “even out”, causing gender differences to disappear.

Gender being an incomplete explanation of the observed differences in teenage e-cigarette use, we draw attention to intersectionality [44] as a promising framework to achieve new understandings of teenage tobacco use. Although intersectionality has been examined in relation to adult smoking cessation (e.g., [45, 46]), there is little or no research to date on teenage tobacco use (and consequent health inequalities) that captures the complexity of “multiple aspects of identity” [41], employing analyses at the intersections of, for example, categories of gender, race, class/SES, disability, sexuality, and religion. More complex conceptual analyses are needed to generate new insights into this emergent and increasing problem of new tobacco product use by young people.

As our findings provide further support for the many studies that have found that peer smoking influences teenage e-cigarette use [20, 47–50], we suggest that health education interventions that take account of peer influences remain important, particularly as higher levels of peer e-cigarette use [51, 52] and favourable e-cigarette peer norms [53] have been found by other researchers to be related to higher odds of personal e-cigarette use.

### Parental monitoring

Parental monitoring was a separately important factor in explaining teenagers’ *e-cigarette use* and we add further support to our previous ESPAD findings [54, 55] - as well as findings from other studies [22, 56] - showing that lack of parental monitoring remains a significant predictor for all illicit substance use in the best-fitting models. However, our finding contrasts with that of Fotiou et al. [49] who reported that low parental monitoring correlates with tobacco but not with e-cigarette use. Our finding about parental monitoring was also gendered, being more significant for teenage boys than for teenage girls.

We highlight an urgent need for health education programmes that address the increasing trend of teenage e-cigarette use and recommend that such programmes acknowledge the important influences of friends and families.

### Limitations of the study

We report on comparable nationally representative samples of teenagers from 2015 and 2019, and note significant gender differences. However, the quantitative methodology does not allow for more in-depth understanding of these gender differences nor why they occur. Thus, in the Discussion section, we offer some possible theories that might be tested in future research to explain the now well-established gender differences in e-cigarette use. Also, longitudinal and/ or qualitative approaches would provide greater insight into teenage e-cigarette use and the associations with the increased risk observed over time.

## Conclusion

Overall, the likelihood of Irish teenagers being e-cigarette users increased significantly between 2015 and 2019. Boys are more likely to be both *ever- and current-users* of e-cigarettes but our trend analyses show that, from one wave to the next, the risk to girls became greater. This differential in rate of increase may reflect differences in how girls and boys are targeted through advertising, gaming and social media platforms, an area requiring further research.

The majority of teenage e-cigarette users had never smoked cigarettes when they first used e-cigarettes, and very few used e-cigarettes as a smoking cessation aid. Rather, the majority used them out of curiosity and, to a lesser extent, because friends did. Our regression model with socio-demographic, personal, peer and familial associations shows that the most prominent risk factors for e-cigarette use were friend and family influences, especially so for boys. In order to support regulation of this rapidly evolving market of new tobacco products, we highlight the role of parental monitoring and peer smoking influences as potential intervention mechanisms for prevention of this increasing addiction to e-cigarettes*.*

## Supplementary Information

**Additional file 1.**

**Additional file 2.**

## Data Availability

All data are available on reasonable request to the corresponding author: lclancy@tri.ie
