# Supplementary material for: Friends and family matter Most: a trend analysis of increasing e-cigarette use among Irish teenagers and socio-demographic, personal, peer and familial associations
Source: BMC Public Health. 2021 Nov 3;21:1988. doi: 10.1186/s12889-021-12113-9 (PMC8567623; doi:10.1186/s12889-021-12113-9)
Supplement: Supplementary file 1 — Additional file 1. [file 12889_2021_12113_MOESM1_ESM.docx]

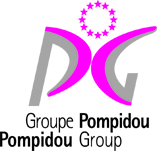

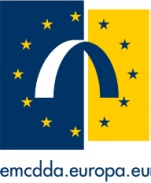


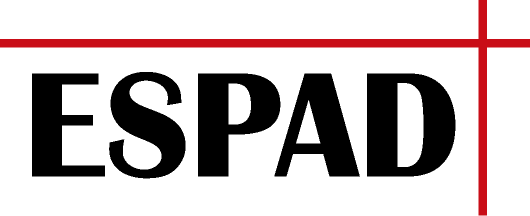


The European School Survey Project on Alcohol and Other Drugs

www.espad.org

## Questionnaire on substance use

**Read this first please!**

This questionnaire is part of an international study on substance use among European students. It will be answered by more than 100,000 students in over 35 countries. The study is called ESPAD.

This is a totally anonymous questionnaire. You should not state your name or any other information which identifies you. You should place your completed questionnaire in the enclosed envelope and seal it yourself. Your teacher will collect the envelopes after completion.

Your class has been randomly selected to take part in this study. In Ireland, the survey is carried out by the TobaccoFree Research Institute Ireland. It is voluntary to take part. If there is any question you don’t want to answer, just leave it blank. It is important that you answer as thoughtfully and honestly as possible. The results will not be presented by single classes and remember your answers are totally anonymous.

If you do not find an answer that fits exactly, indicate the one that comes closest. Please, mark the appropriate answer to each question by making an "X" in the box. If you have a question, please raise your hand and your teacher will assist you.

**Thank you in advance for your participation! Please begin.**


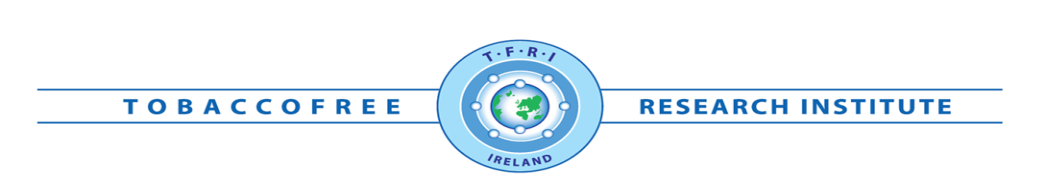


**TobaccoFree Research Institute Ireland**

D.I.T. Focas Building 8 Camden Row, Dublin 8.

Tel 353 1 5388372 Email [lclancy@tri.ie](mailto:lclancy@tri.ie); [skeogan@tri.ie](mailto:skeogan@tri.ie); [kbabineau@tri.ie](mailto:kbabineau@tri.ie)

[www.tri.ie](http://www.tri.ie)

**The first questions ask for some background information about yourself and the kinds of things you might do**

**The first questions ask for some background information about yourself and the kinds of things you might do**

**C01**

**What is your sex?**

1  Male

2  Female

**C02**

**When were you born?**

(Mark 01 for January, 02 for February …

Month * . … and 12 for December)

Year 19

**C03**

How often (if at all) do you do each of the following?

Mark one box for each line.

A few Once or At least Almost

times a twice a once a every

Never year month week day

a) Play computer games

b) Actively participate in sports, athletics or exercising

c) Read books for enjoyment (do not count schoolbooks)

d) Go out in the evening (to a disco, cafe, party etc)

e) Other hobbies (play an instrument, sing, draw, write)

f) Go around with friends to shopping centres, streets, parks etc just for fun

g) Use the Internet for leisure activities (chats, music, games, social

networks, videos etc)

h) Play on slot machines (the kind in which you may win money)

1 2 3 4 5

**C04**

During the LAST 30 DAYS on how many days have you missed one or more lessons?

Mark one box for each line.

7 days
 None 1 day 2 days 3–4 days 5–6 days or more

a) Because of illness

b) Because you skipped or ”cut”

c) For other reasons

1 2 3 4 5 6

**The following questions are about cigarette smoking**

**C056**

How difficult do you think it would be for you to get cigarettes if you wanted?

1  Impossible

2  Very difficult

3  Fairly difficult

4  Fairly easy

5  Very easy

6  Don’t know

**C06**

**On how many occasions (if any) during your lifetime have you smoked cigarettes?**

Number of occasions

0 1–2 3–5 6–9 10–19 20–39 40 or more

1 2 3 4 5 6 7

**C07**

**How frequently have you smoked cigarettes during the LAST 30 DAYS?**

1  Not at all

2  Less than 1 cigarette per week

3  Less than 1 cigarette per day

4  1–5 cigarettes per day

5  6–10 cigarettes per day

6  11–20 cigarettes per day

7  More than 20 cigarettes per day

**C08**

**When (if ever) did you FIRST do each of the following things?**

Mark one box for each line.

9 years 10 11 12 13 14 15 16

old or years years years years years years years

Never less old old old old old old or older

a) Smoke your first cigarette
b) Smoke cigarettes on a daily basis

1 2 3 4 5 6 7 8 9

**The next questions are about alcoholic beverages**

**– including beer, cider, alcopops (premixed drinks), wine and spirits**

**C09**

How difficult do you think it would be for you to get each of the following, if you wanted?

Mark one box for each line.

Impos- Very Fairly Fairly Very Don’t

sible difficult difficult easy easy know

a) Beer

b) Cider

c) Alcopops

d) Wine

e) Spirits

1 2 3 4 5 6

**C10**

On how many occasions (if any) have you had any alcoholic beverage to drink?

Mark one box for each line.

Number of occasions

40 or

0 1–2 3–5 6–9 10–19 20–39 more

a) In your lifetime

b) During the last 12 months
c) During the last 30 days

1 2 3 4 5 6 7

**Think back over the LAST 30 DAYS. On how many occasions (if any) have you had any of the**

**C11**

**following to drink?**

Mark one box for each line.

Number of occasions

40 or

0 1–2 3–5 6–9 10–19 20–39 more

a) Beer

b) Cider

c) Alcopops

d) Wine

e) Spirits

1 2 3 4 5 6 7

**The following questions are about the last day you drank alcohol**

**C12**

**When was the last day you drank alcohol?**

1  I never drink alcohol

2  1–7 days ago

3  8–14 days ago

4  15–30 days ago

5  1 month – 1 year ago

6  More than 1 year ago

**C13**

**Think of the LAST DAY that you drank any alcohol. Which of the following beverages did you drink**

**on that day?**

Mark all that apply.

1  I never drink alcohol

1  Beer

1  Cider

1  Alcopops

1  Wine

1  Spirits

**C13a If you drank beer that last day you drank any**

**alcohol, how much did you drink?**

1  I never drink beer

2  I did not drink beer on the last day

that I drank alcohol

3  Less than one regular bottle or can

4  1-2 regular bottles or cans

5  3-4 regular bottles or cans

6  More than 4 regular bottles or cans

**OC13b If you drank cider that last day you drank any**

alcohol, how much did you drink? *

1  I never drink cider

2  I did not drink cider on the last day

that I drank alcohol

3  Less than one regular bottle or can

4  1-2 regular bottles or cans

5  3-4 regular bottles or cans

6  More than 4 regular bottles or cans

OC13c If you drank alcopops that last day you drank

any alcohol, how much did you drink? *

1  I never drink alcopops

2  I did not drink alcopops on the last day

that I drank alcohol

3  Less than one regular bottle or can

4  1-2 regular bottles or cans

5  3-4 regular bottles or cans

6  More than 4 regular bottles or cans

**C13d If you drank wine that last day you drank any alcohol, how much did you drink?**

1  I never drink wine

2  I did not drink wine on the last day

Heavily intoxicated, for example not remembering

what happened

that I drank alcohol

3  Less than 2 glasses

4  2-3 glasses (about half a bottle)

Not at all

5  4-6 glasses

6  6+ glasses (a bottle of wine or more)

**C13e If you drank spirits that last day you drank any alcohol, how much did you drink?**

1  I never drink spirits

2  I did not drink spirits on the last day

that I drank alcohol

3  Less than 2 drinks

4  2-3 drinks

5  4-6 drinks

6  More than 6 drinks

**C13f Please indicate on this scale from 1 to 10 how**

**drunk you would say you were that last day you**

**drank alcohol. (If you felt no effect at all you**

**should mark “1”.)**

Heavily intoxicated, for

example not remembering

what happened

Not at all

1 2 3 4 5 6 7 8 9 10

I never drink alcohol

11

**The next question is about alcohol consumption during the last 30 days**

**C14**

Think **Think back again over the LAST 30 DAYS. How many times (if any) have you had five or more drinks**

on one occasion? (A ”drink” is a glass/bottle/can of beer, a bottle/can of cider, 2 glasses/bottles of alcopops, a glass of wine, a glass of spirits or a mixed drink].)

1  None

2  1

3  2

4  3–5

5  6–9

6  10 or more times

**The next couple of questions are also about alcohol**

**C158**

**On how many occasions (if any) have you been intoxicated from drinking alcoholic beverages, for**

**example staggered when walking, not being able to speak properly, throwing up or not remembering**

**what happened?**

Mark one box for each line.

Number of occasions

40 or

0 1–2 3–5 6–9 10–19 20–39 more

a) In your lifetime

b) During the last 12 months
c) During the last 30 days

1 2 3 4 5 6 7

**C16**

**When (if ever) did you FIRST do each of the following things?**

Mark one box for each line.

9 years 10 11 12 13 14 15 16

old or years years years years years years years

Never less old old old old old old or older

a) Drink beer (at least one glass)
b) Drink cider (at least one glass)
c) Drink alcopops (at least one glass)
d) Drink wine (at least one glass)
e) Drink spirits (at least one glass)
f) Get drunk on alcohol

1 2 3 4 5 6 7 8 9

**C17**

**WHILE UNDER THE INFLUENCE OF ALCOHOL, how often during the LAST 12 MONTHS have you**

experienced the following?

Mark one box for each line.

I have not drunk any alcohol during the last 12 months Please continue with question C18

Number of occasions

40 or

0 1–2 3–5 6–9 10–19 20–39 more

a) Physical fight

b) Accident or injury

c) Damaged or lost objects or clothing

d) Serious arguments

e) Victimized by robbery or theft

f) Trouble with police

g) Hospitalised or admitted to an emergency room because of

severe intoxication        h) Hospitalised or admitted to an emergency room because of

accident or injury        i) Engaged in sexual intercourse without a condom        j) Being a victim of unwanted sexual advance

k) Deliberately hurt yourself

l) Driven a moped, car or other motor vehicle

m) Being involved in an accident while driving yourself

n) Been swimming in deep water (swimming pool, river, lake

or sea)

1 2 3 4 5 6 7

**C18**

**Have you experienced problems during the LAST 12 MONTHS that occurred because of someone**

**else´s drinking?**

Mark one or more boxes for each line

No Yes, a Yes, a Yes, some-

stranger friend or body else

acquain- close to me

tance

a) Has someone who had been drinking harassed or bothered you at a party or

some other private setting?

b) Has someone who had been drinking harassed or bothered you on the street

or in some public place?

c) Has someone who had been drinking harmed you physically?

d) Has someone who had been drinking ruined your clothes or other belongings?

e) Has someone who has been drinking been responsible for a traffic accident you

were involved in?

f) Have you been a passenger with a driver who had had too much to drink?

g) Has someone who had been drinking made you afraid when you encountered

them on the street?

1 1 1 1

**C19**

In your view, does a person close to you drink excessively?

1  No

2  Yes Has this caused harm or problems in your life?

1  No

**2**  Yes

**Tranquillisers and sedatives, like benzos or tablets, are sometimes prescribed by doctors to help people to calm down, get to sleep or to relax. Pharmacies are not supposed to sell them without a prescription.**

**C20**

**Have you ever taken tranquillisers or sedatives because a doctor told you to take them?**

1  No, never

2  Yes, but for less than 3 weeks

3  Yes, for 3 weeks or more

**The next questions ask about marijuana or hashish (cannabis)**

**C21**

How difficult do you think it would be for you to get marijuana or hashish (cannabis) if you wanted?

1  Impossible 4  Fairly easy

2  Very difficult 5  Very easy

3  Fairly difficult 6  Don’t know

**C22**

**On how many occasions (if any) have you used marijuana or hashish (cannabis)?**

Mark one box for each line.

Number of occasions

40 or

0 1–2 3–5 6–9 10–19 20–39 more

a) In your lifetime

b) During the last 12 months
c) During the last 30 days

1 2 3 4 5 6 7

**C23**

When (if ever) did you FIRST try marijuana or hashish (cannabis)?

1  Never 6  13 years old

2  9 years old or less 7  14 years old

3  10 years old 8  15 years old
 4  11 years old 9  16 years or older

5  12 years old

**C24**

Have you ever had the possibility to try marijuana or hashish (cannabis) without trying it?

1  No

2  Yes How many times has this happened in your life?

1  1–2

2  3–5

3  6–9

4  10–19

5  20–39

6  40 or more

**The next questions ask about some other drugs**

**C25**

How difficult do you think it would be for you to get each of the following, if you wanted?

Mark one box for each line.

Very Fairly Fairly Very Don’t

Impossible difficult difficult easy easy know

a) Amphetamines (speed, uppers)

b) Methamphetamines (crystal meth)

c) Tranquillisers or sedatives (benzos, tablets)       d) Ecstasy (MDMA, Molly)

e) Cocaine (coke)

f) Crack

g) Heroin (gear)

1 2 3 4 5 6

**C26**

**On On how many occasions (if any) have you used ecstasy (MDMA, Molly)?**

Mark one box for each line.

Number of occasions

40 or

0 1–2 3–5 6–9 10–19 20–39 more

a) In your lifetime

b) During the last 12 months
 1 2 3 4 5 6 7

**C27**

On how many occasions (if any) have you used amphetamines (speed, uppers)?

Mark one box for each line.

Number of occasions

40 or

0 1–2 3–5 6–9 10–19 20–39 more

a) In your lifetime

b) During the last 12 months
 1 2 3 4 5 6 7

**C28**

On how many occasions (if any) have you used methamphetamines (meth, crystal)?

Mark one box for each line.

Number of occasions

40 or

0 1–2 3–5 6–9 10–19 20–39 more

a) In your lifetime

b) During the last 12 months
 1 2 3 4 5 6 7

**C29**

On how many occasions (if any) have you used cocaine?

Mark one box for each line.

Number of occasions

40 or

0 1–2 3–5 6–9 10–19 20–39 more

a) In your lifetime

b) During the last 12 months

1 2 3 4 5 6 7

**C30**

On how many occasions (if any) have you used crack?

Mark one box for each line.

Number of occasions

40 or

0 1–2 3–5 6–9 10–19 20–39 more

a) In your lifetime

b) During the last 12 months
 1 2 3 4 5 6 7

**C31**

On how many occasions (if any) have you used inhalants [glue, aerosol, paint] to get high?

Mark one box for each line.

Number of occasions

40 or

0 1–2 3–5 6–9 10–19 20–39 more

a) In your lifetime

b) During the last 12 months
c) During the last 30 days

1 2 3 4 5 6 7

**C32**

On how many occasions in your lifetime (if any) have you used any of the following drugs?

Mark one box for each line.

Number of occasions

40 or

0 1–2 3–5 6–9 10–19 20–39 more

a) Tranquillisers or sedatives (without a doctor’s prescription)

b) LSD or some other hallucinogens

c) Relevin

d) Heroin

e) ”Magic mushrooms”

f) GHB

g) Anabolic steroids

h) Drugs by injection with a needle (like heroin, cocaine,

amphetamine, steroids)

i) Alcohol together with pills (medicaments) in order to get high

j) Painkillers in order to get high ……………………………………….

**C33**

**When (if ever) did you FIRST do each of the following things?**

Mark one box for each line.

9 years 10 11 12 13 14 15 16

old or years years years years years years years

Never less old old old old old old or older

a) Try tranquillisers or sedatives (without

a doctor’s prescription)
 b) Try amphetamines or methamphetamines

c) Try cocaine or crack

d) Try ecstasy, MDMA

e) Try inhalants (glue, aerosol, paint) in

order to get high

f) Try alcohol together with pills (medica-

ments) in order to get high

1 2 3 4 5 6 7 8 9

**The next questions ask about new substances**

**C34**

New **New substances that imitate the effects of illicit drugs [such as cannabis or ecstasy] may now be**

**sometimes available. They are sometimes called [‘legal highs’, ‘ethno botanicals’, ‘research**

**chemicals’] and can come in different forms, for example – herbal mixtures, powders, crystals or**

**tablets.**

**Have you ever used such substances?**

1  Yes, I have used such substances

2  No, I never used such substances

3  Don’t know/ Not sure

**C35**

What was the appearance/form of the new substance you used in the LAST 12 MONTHS?

Mark one or more boxes.

1  I have not used such substances in the last 12 months

1  Herbal smoking mixtures with drug-like effects

1  Powders, crystals or tablets with drug-like effects

1  Liquids with drug-like effects

1  Other

**The next questions ask about various substances**

**C36**

How How much do you think PEOPLE RISK harming themselves (physically or in other ways), if they …

Mark one box for each line.

No risk Slight Moderate Great Don’t

risk risk risk know

a) smoke cigarettes occasionally

b) smoke one or more packs of cigarettes per day

c) have one or two drinks nearly every day

d) have four or five drinks nearly every day

e) have five or more drinks in one occasion nearly each weekend

f) try marijuana or hashish (cannabis) once or twice

g) smoke marijuana or hashish (cannabis) occasionally

h) smoke marijuana or hashish (cannabis) regularly

i) try ecstasy once or twice

j) take ecstasy regularly

k) try an amphetamine (uppers, pep pills, bennie, speed) once or twice

l) take amphetamines regularly

1 2 3 4 5

**The next questions ask about Internet, gaming and gambling**

**C37**

##### During the LAST 7 DAYS, which days (if any) were you on the Internet (on a computer, tablet,

##### smartphone, console or any other electronic device)? Please include all kinds of Internet activities.

Mark one or more boxes.

None Monday Tuesday Wednesday Thursday Friday Saturday Sunday

1 1 1 1 1 1 1 1

**C38**

##### C4 During the LAST 7 DAYS, how many hours (if any) were you on the Internet (on a computer, tablet,

##### smartphone, console or any other electronic device) on a TYPICAL WEEKDAY and a TYPICAL

##### WEEKEND DAY? Please include all kinds of Internet activities.

Mark one box for each line.

None Half an hour About 1 About 2-3 About 4-5 6 hours

or less hour hours hours or more

a) Typical weekday (Monday-Thursday)

b) Typical weekend day (Friday-Sunday)

1 2 3 4 5 6

**C39**

##### During the LAST 7 DAYS, on how many days (if any) were you on the Internet?

Mark one box for each line.

None 1 day 2 days 3 days 4 days 5 days 6 days 7 days

a) On Social Media (communicating with others on the

Internet, using for example WhatsApp, Twitter, Facebook,

Skype, Blogs, Snapchat, Instagram, etc)

b) Playing online games (war, strategy and first-person

shooter games, World of Warcraft, Call of Duty, Grand

Theft Auto, MMO, MMORPG etc)

c) Playing games in which you may win money (poker,

scratch, dice, new slot etc)

d) Reading, surfing, searching for information etc

e) Streaming/downloading music, videos, films etc         f) Searching for, selling or buying products, games,

books etc (Amazon, Ebay etc) 1 2 3 4 5 6 7 8

**C40**

##### During the LAST 30 DAYS, how many hours (if any) did you spend on the Internet on a TYPICAL

##### DAY?

##### Mark one box for each line.

None Half an hour About 1 About 2-3 About 4-5 6 hours

or less hour hours hours or more

a) On Social Media (communicating with others on the Internet, using for

example WhatsApp, Twitter, Facebook, Skype, Blogs, Snapchat,

Instagram, Kik etc)

b) Playing online games (war, strategy and first-person shooter games,

World of War craft, Call of Duty, Grand Theft Auto, MMO, MMORPG etc)

c) Playing games in which you may win money (poker, scratch, dice,

new slot etc)

d) Reading, surfing, searching for information etc

e) Streaming/downloading music, videos, films etc

f) Searching for, selling or buying products, games, books etc

[Amazon, Ebay etc]

1 2 3 4 5 6

**C41**

**How much do you agree or disagree with the following statements on Social Media**

**(communicating with others on the Internet, using for example WhatsAapp, Twitter, Facebook,**

**Skype, Blogs, Snapchat, Instagram etc).**

Mark one box for each line.

Strongly Partly Neither Partly Strongly

agree agree nor disagree disagree

a) I think I spend way too much time on Social Media

b) I get in bad mood when I cannot spend time on Social Media      c) My parents say that I spend way too much time on Social Media

1 2 3 4 5

**C42**

How **How much do you agree or disagree with the following statements about gaming on a computer,**

**tablet, console, smartphone or other electronic device?**

Mark one box for each line.

Strongly Partly Neither Partly Strongly

agree agree nor disagree disagree

a) I think I spend way too much time playing games

b) I get in bad mood when I cannot spend time on games      c) My parents say that I spend way too much time on gaming

1 2 3 4 5

**C43**

**H How often (if ever) did you gamble money in the LAST 12 MONTHS?**

1  I have not gambled money during the last 12 months

2  Monthly or less

3  2-4 times a month

4  2-3 times a week

5  4-5 times a week

6  6 or more times a week

**C44**

If you have gambled money in the LAST 12 MONTHS, which games have you played ON THE

INTERNET?

Mark one box for each line.

I have not Monthly 2-4 times 2-3 times 4-5 time 6 or more

played or less a months a week a week times a

these week

games

a) Slot machines (fruit machine, new slot etc)

b) Play card or dice (poker, bridge, dice etc)

c) Lotteries (scratch, bingo, keno etc)       d) Betting on sports or animals (horses, dogs etc)

1 2 3 4 5 6

**C45**

**If you have gambled money in the LAST 12 MONTHS, which games have you played** **NOT ON**

**THE INTERNET (in traditional settings)?**

Mark one box for each line.

I have not Monthly 2-4 times 2-3 times 4-5 time 6 or more

played or less a months a week a week times a

these week

games

a) Slot machines (fruit machine, new slot etc)

b) Play card or dice (poker, bridge, dice etc)
 c) Lotteries (scratch, bingo, keno etc)

d) Betting on sports or animals (horses, dogs etc)

1 2 3 4 5 6

**The next questions ask about your parents. If mostly foster parents, step-parents or others brought you up answer for them. For example, if you have both a stepfather and a natural father, answer for the one that is the most important in bringing you up**

**C46**

I**n which country were you and your parents born?**

Mark one box for each line.

Ireland UK Poland Nigeria Lithuania Other country (please write in)

a) Yourself       __________

b) Your mother      …………….__________

c) Your father      …………….__________

1 2 3 4 5 6

**Are you ever been treated badly or unfairly because of your skin colour, ethnicity, religion, or birth country?**

Mark one or more boxes.

1  Everyday

1  On a weekly basis

1  On a monthly basis

1  It has happened once or twice

1  Never

**C47**

What is the highest level of schooling your father completed?

1  Completed primary school or less

2  Some secondary school

3  Completed secondary school

4  Some college or university

5  Completed college or university

6  Don't know

7  Does not apply

**C48**

What is the highest level of schooling your mother completed?

1  Completed primary school or less

2  Some secondary school

3  Completed secondary school

4  Some college or university

5  Completed college or university

6  Don't know

7  Does not apply

**C49**

**How well off is your family compared to other families in your country?**

1  Very much better off

2  Much better off

3  Better off

4  About the same

5  Less well off

6  Much less well off

7  Very much less well off

**C50**

**Which of the following people live in the same household with you?**

Mark all that apply.

1  I live alone 1  Brother(s)

1  Father 1  Sister(s)

1  Stepfather 1  Grandparent(s)

1  Mother 1  Other relative(s)

1  Stepmother 1  Non-relative(s)

**C5119**

**How often do the following statements apply to you?**

Mark one box for each line.

Almost Some- Almost

always Often times Seldom never

a) My parent(s) set definite rules about what I can do at home

b) My parent(s) set definite rules about what I can do outside the home

c) My parent(s) know whom I am with in the evenings

d) My parent(s) know where I am in the evenings

e) I can easily get warmth and caring from my mother and/or father

f) I can easily get emotional support from my mother and/or father

g) I can easily borrow money from my mother and/or father

h) I can easily get money as a gift from my mother and/or father

i) I can easily get warmth and caring from my best friend

j) I can easily get emotional support from my best friend

1 2 3 4 5

**C52**

Do your parents know where you spend Saturday nights?

1  Know always

2  Know quite often

3  Know sometimes

4  Usually don’t know

**C53**

**If you had ever used marijuana or hashish (cannabis), do you think that you would have said so in**

**this questionnaire?**

1  I already said that I have used it

2  Definitely yes

3  Probably yes

4  Probably not

5  Definitely not

**This section includes some more questions about cannabis**

**MA1**

Have you used cannabis during the LAST 12 MONTHS?

1  No

2  Yes Has the following happened to you during the LAST 12 MONTHS?

Mark one box for each line.

From time Fairly Very

Never Rarely to time often often

a) Have you smoked cannabis before midday?

b) Have you smoked cannabis when you were alone?

c) Have you had memory problems when you smoked cannabis?

d) Have friends or members of your family told you that you

ought to reduce or stop your cannabis use?

e) Have you tried to reduce or stop your cannabis use with-

out succeeding?

f) Have you had problems because of your use of cannabis

(argument, fight, accident, bad result at school, etc)?

Which:

1 2 3 4 5

**MA2**

**Are you part of a clique of friends, where using cannabis is part of your behaviour when you meet?**

1  No

2  Yes How often per month do you meet with members of this clique?

1  (Almost) daily

2  3–4 times a week

3  1–2 times a week

4  1–3 times a month

5  Less than once a month

**The next questions are about yourself and what you think about others**

**O01**

**Which of the following best describes your average grade at the end of the last term?**

1  A (100%-85%)

2  B (84%-70%)

1  C (69%-55%)

2  D (54%-40%)

1  F (Lower than 40%)

**O02**

How satisfied are you usually with …

Mark one box for each line.

Very Neither Not so Not at all There is no

satisfied Satisfied nor satisfied satisfied such person

a) your relationship with your mother?

b) your relationship with your father?

c) your relationship with your friends?

1 2 3 4 5 6

**O03**

**What do you think your mother’s reaction would be if you do the following things?**

Mark one box for each line.

She would She would She would She would

not allow it discourage it not mind approve of it Don’t know

a) Get drunk

b) Use marijuana/hashish

c) Use ecstasy

1 2 3 4 5

**O044**

**What do you think your father’s reaction would be if you do the following things?**

Mark one box for each line.

He would He would He would He would

not allow it discourage it not mind approve of it Don’t know

a) Get drunk

b) Use marijuana/hashish

c) Use ecstasy

1 2 3 4 5

**O05**

How many of your friends would you estimate…

Mark one box for each line.

None A few Some Most All

a) smoke cigarettes

b) drink alcoholic beverages (beer, cider, alcopops, wine, spirits)

c) get drunk

d) smoke marijuana or hashish (cannabis)

e) take tranquillisers or sedatives (without a doctor’s prescription)

f) take ecstasy

g) use inhalants

1 2 3 4 5

**Now follow another few questions about smoking and tobacco**

**O06**

**Have you ever used e-cigarettes or water pipe?**

Mark one box for each line.

Yes, in the Yes, in the Yes, but more Never

last 30 days last 12 months than 12

months ago

a) Water pipe

b) E- cigarettes

**O07**

**When (if ever) did you FIRST do each of the following things?**

Mark one box for each line.

9 years 10 11 12 13 14 15 16

old or years years years years years years years

Never less old old old old old old or older

a) Use your first e-cigarette
b) Use e-cigarettes on a daily basis

1 2 3 4 5 6 7 8 9

**I01 Why did you try e-cigarettes for the first time?**

Tick all that apply

a) to try to quit smoking tobacco

b) as an alternative to smoking tobacco

c) because your friends were using e-cigarettes

d) because you were curious

e) I don’t know

f) I’ve never tried e-cigarettes…………………………...…………………………..

e) I’ve never tried e-cigarettes

I02 When you first tried an e-cigarette, what was your relationship with tobacco?

a) I had never smoked tobacco

b) I had tried tobacco but didn’t use it regularly

c) I smoked tobacco occasionally

d) I smoked tobacco regularly

e) I’ve never tried an e-cigarette

**The next questions ask once more about alcohol**

**O09 Think back over the LAST 30 DAYS. On how many occasions (if any) have you bought beer, cider,**

**alcopops, wine or spirits in a shop (grocery store, off license, or petrol station) for your own**

**consumption (off-premise)?**

Mark one box for each line.

Number of occasions

20 or

0 1–2 3–5 6–9 10–19 more

a) Beer

b) Cider

c) Alcopops
d) Wine

e) Spirits

1 2 3 4 5 6

**O10**

**Think back once more over the LAST 30 DAYS. On how many occasions (if any) have you drunk**

**beer, cider, alcopops, wine or spirits in a pub, bar, restaurant or disco (on-premise)?**

Mark one box for each line.

Number of occasions

20 or

0 1–2 3–5 6–9 10–19 more

a) Beer

b) Cider

c) Alcopops

d) Wine

e) Spirits

1 2 3 4 5 6

**O11**

**Think of that last day on which you drank alcohol. Where were you when you drank?**

Mark all that apply.

1  I never drink alcohol

1  At home

1  At someone else's home

1  Out on the street, in a park, beach or other open area

1  At a bar or a pub

1  In a disco

1  In a restaurant

1  Other places (please describe) …………………………………………………………………....................................…………….

**O12**

In the LAST 12 MONTHS, how often did you drink …

Mark one box for each line.

Never Seldom Sometimes Mostly Always

a) because it helps you enjoy a party

b) because it helps you when you feel depressed or nervous

c) to cheer up when you're in a bad mood

d) because you like the feeling

e) to get high

f) because it makes social gatherings more fun

g) to fit in with a group you like

h) because it improves parties and celebrations

i) to forget about your problems

j) because it's fun

k) to be liked

l) so you won´t feel left out ……

1 2 3 4 5
